# Parametric Optimization of Trochoidal Step on Surface Roughness and Dish Angle in End Milling of AISID3 Steel Using Precise Measurements

**DOI:** 10.3390/ma12081335

**Published:** 2019-04-24

**Authors:** Santhakumar J, Mohammed Iqbal U

**Affiliations:** Department of Mechanical Engineering, Faculty of Engineering and Technology, SRM Institute of Science and Technology, Kattankulathur, Tamil Nadu 603203, India; jsanthakumar1987@gmail.com

**Keywords:** dish angle, trochoidal step, response surface methodology, surface roughness, desirability approach

## Abstract

Tool steel play a vital role in modern manufacturing industries due to its excellent properties. AISI D3 is a cold work tool steel which possess high strength, more hardenability and good wear resistance properties. It has a wide variety of applications in automobile and tool and die making industries such as blanking and forming tools, high stressed cutting, deep drawing and press tools. The novel ways of machining these steels and finding out the optimum process parameters to yield good output is of practical importance in the field of research. This research work explores an attempt to identify the optimized process parameter combinations in end milling of AISI D3 steel to yield low surface roughness and maximum dish angle using trochoidal milling tool path, which is considered as a novelty in this study. 20 experimental trials based on face centered central composite design (CCD) of response surface methodology (RSM) were executed by varying the input process factors such as cutting speed, feed rate and trochoidal step. Analysis of variance (ANOVA) was adopted to study the significance of selected process parameters and its relative interactions on the performance measures. Desirability-based multiple objective optimization was performed and the mathematical models were developed for prediction purposes. The developed mathematical model was statistically significant with optimum conditions of cutting speed of 41m/min, feed rate of 120 mm/min and trochoidal step of 0.9 mm. It was also found that the deviation between the experimental and predicted values is 6.10% for surface roughness and 1.33% for dish angle, respectively.

## 1. Introduction

**Figure 1:**
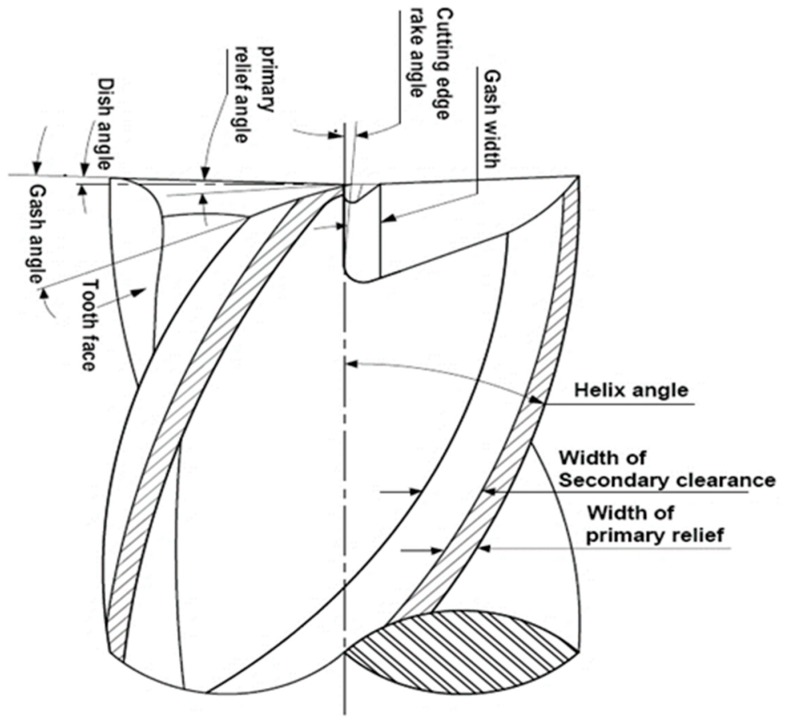
Geometrical parameters of an end mill.

The overall machining economy, performance and efficiency are largely affected by the cutting tool and its geometry. Cutting tools generally fail due to rapid plastic deformation or mechanical breakage at the cutting edges and often because of gradual wear [1,2]. Failures due to plastic deformation and sudden breakage have considerable adverse effect. Generally, the helical end mills having two or more flutes are widely utilized for slotting, facing, profiling and grooving of narrow surfaces. The end mill cutter edges accomplish the foremost cutting work and generate the flow surface profile at the time of machining process. Dish angle is one of most significant parameters of cutting tool edge which ensures that the flat surface is produced by the cutter during machining. Angle formed between end cutting edge and perpendicular to plane of the cutter axis is known as dish angle as shown in Figure 1. During milling operation, the cutting tool is subjected to wear; as a result, the dish angle value decreases, which influence the more engagement length between workpiece and tool during machining. Thus, the height of residual ridge is increased, which leads to poor surface finish. Therefore, a cutting tool with dish angle is required to achieve a moderate surface quality and tool life [3,4].

The quality of the machined surface is an essential parameter to assess the productivity of machine tools and the machined components. Therefore, obtaining the required surface quality is vital to the functional behavior aspects of mechanical parts [5]. Hence, it is required to assess the important parameter that affects the quality characteristic of the machined surface. Not only process parameter, but also geometric parameters such as relief angle, gash angle, gash rake angle, helix angle, pitch angle and fluting rake angle difference influence the surface roughness on machined surfaces [6,7]. 

In many manufacturing sectors, end milling operation is most utilizing processes for making the variety of components such as die and mold [8]. Milling of these components is performed by employing different tool paths such as linear and non-linear tool path strategies [9]. The implementation of traditional tool paths during high speed machining has induced effects such as cutting forces, machining vibrations, damage to the tool and precision loss. To eliminate these effects, many researches have started focusing on the trochoidal milling, which is an amalgamation of circular and linear motions with simultaneous execution. Trochoidal milling is a promising tool path, which can reduce the sudden change of the dynamic cutting force due to the continuous change of radial depth of cut [10]. This is one of the most utilizing tool paths for machining narrow slot and other complex cavities. The major concern in this tool path is the large trochoidal tool path step parameter which leads to increase the cutting force and tool wear resulting in poor surface quality, while the conservative trochoidal step restricts the efficiency of machining [11,12]. Therefore, it is required to analyze and predict the effect of tool path parameter in order to yield the better machining performance in trochoidal milling. In recent years, certain commercial computer aided manufacturing (CAM) software has inbuilt function of trochoidal machining methods. Many researchers have also propelled corresponding studies on trochoidal machining. 

Deng et al. [13] have proposed a method to choose optimal trochoidal tool path parameters to minimize the machining time and have analyzed the relationship between MRR and cutting forces. Lopez de lacalle et al. [14] developed modeling of the end milling process to estimate the real tool-path and the tool deflection on cutting force. Modifications were made in the model for minimum chip thickness influence and its relationship with the large tool deflection and the effect of tool cutting edge radius with respect to coefficients of cutting force. It was reported that consistency of simulated cutting force and measured cutting force are in good agreement during end milling process. Patil et al. [15] implemented the trochoidal tool path strategy on Ti6Al4V to study its impact on quality and productivity, and concluded that the tool path generates better surface roughness and tool life. Shixiong [16] studied the trochoidal machining on high speed pocket milling operation and it was reported that path geometry has to be optimized to reduce the trochoidal tool path length feed rate and depth of cut, and the other parameters have to be increased within a bearing capability of tool which enhances the machining efficiency.

Generally, the quality of the end milled components depend upon the other machining factors such as depth of cut, feed rate, cutting speed etc. [17]. Therefore, there is a need of optimizing the end mill parameters in addition to trochoidal tool path parameter required for the better machining performance characteristics. Conventionally, many trials were performed for the selection of end milling process parameters but it was time consuming and costlier. Hence, it is required to develop a multiple objective optimization methodology that can predict the output responses for reducing the surface roughness and maximize the dish angle of end milling process.

Many researchers have reported on multi-response optimization in the end milling process. Sivaraosa et al. [18] stated that the Response Surface Methodology (RSM) approach is an effective technique by its precision towards experimental validation and mathematical modeling. The prominence of an interactive term and square term of factors are accurately predicted by RSM. Two-dimensional (2D) contour and three-dimensional (3D) surface plot developed by RSM helped in identifying the interactive influence of factors on output responses within the range of specified limits. Mia [19] adopted the RSM technique for developing mathematical modeling, in which the optimization was attained by the composite desirability function on the cutting force, surface roughness and specific cutting energy in slot milling operation using AISI 1060 steel performed through-tool cryogenic cooling condition. Similarly, Abou-El-Hossein et al. [20] developed a 1st and 2nd order model for cutting force that has been generated during the end milling process of modified AISI P20 tool steel using RSM approach. The effect of cutting speed, axial depth of cut, feed rate and stepover on cutting force was stated. It was observed that there is a strong interaction among feed rate and axial depth and a quite significant interaction of feed rate and stepover. Calıskan et al. [21] studied the effect of various coating tools on surface roughness and cutting force using RSM technique in the face milling operation of hardened steel. It was concluded that an interaction of different coating type of tools and depth of cut influence the surface roughness, whereas cutting force was not affected by hard coating tool.

From the literature studies, it was analyzed that the researchers have investigated the effects of trochoidal machining extensively without considering the influence of trochoidal step parameter on surface roughness and dish angle. The purpose of the present investigation was to employ experimental and statistical methods to examine the role of process parameters on the surface roughness and dish angle in AISI D3 cold work tool steels using trochoidal tool path. Novel parameters such as trochoidal step and dish angle are taken as an input and output responses for this study. Predictive modeling for the input variable such as cutting speed, feed rate and trochoidal step combinations in end milling is another aspect that has not been explored by researchers. To investigate the effect of output responses with respect to input variable, the RSM technique is well suited. Hence, RSM-based desirability multi-objective optimization approach was employed to arrive the optimal solution during end milling process. 

The layout of this article is composed of various sections in which the first section outlines the gaps identified in the extensive literature studies, need and objectives of the proposed research. Section two deals with the materials and methodology of this research. It elaborates the importance of AISI D3 applications and its properties, selection of process parameter and simulation of trochoidal step increment followed by cutting tool calibration measurement technique on dish angle and surface roughness. In the third section, the design of the experiments’ orientation plan was formed with the input factors based on central composite design (CCD) for formulating the estimation models with respect to dependent factors and evaluating the adequacy of the model based on analysis of variance (ANOVA) was discussed. The fourth section elaborates the effect of each output responses, using 3D RSM surface plots and determining the optimum process parameters using desirability based multi objective optimization. The last section describes the conclusion made from the investigation.

## 2. Materials and Methods

The material utilized for this study is AISI D3 cold work tool steel (Tradewell Ferromet Private Limited, Mumbai, India), of which the chemical composition in wt% and its mechanical properties are shown in Table 1 and Table 2 respectively. It is widely utilized in the production of cold forming dies and molds due to its excellent wear resistance from gliding contact with distinct metals and deep-hardening characteristics for automobile and aerospace applications. End milling operation were executed on a BFW Gaurav BMV 35 T12 (3-AxisVMC, Bharat Fritz Werner Limited, Bengaluru, India) in built with Siemens Sinumerik 828D Basic controller. The traverse xyz axis is 450 × 350 × 350 mm, respectively. The maximum spindle speed and feed rate is 8000 rpm and 10000 mm/min, respectively with spindle motor has a power of 3.7 kW. The machine positioning accuracy and repeatability; accuracy is ±0.005 mm and ±0.003 mm, respectively. All the experiments were performed under dry conditions. The slot size was cut into 35 × 10 mm cross section and 75 mm in length to conduct the experiments and overall 22,500 mm^3^ volume of materials was removed at each experiment. Table 3 illustrates the list of process parameters and its levels used in this study for machining AISID3 steel. The cutting parameters are selected based on the pilot experiments conducted above and below the selected ranges of input parameter from Table 3, cutting speed of 60 m/min, feed rate 450 mm/min and loop spacing 2.5 mm. After conducting the pilot experiments, the machined surface and cutting tool was inspected using surface roughness testing machine and Zoller presetter. It was found that surface roughness value increased above 1 μm due to high feed marks presented on the surfaces and also tooltip got damaged. There was no considerable effect of wear noticed with the experiments conducted below the lower level of the parameters as shown in Table 3. Thus, feasible working limits of cutting speed was safely selected in the range of 15–45 mm/min, feed rate in the range of 120 to 360mm/min and loop spacing 0.6 mm to 1.8 mm for end milling operation. 20 end milling experiment trials based on the CCD of RSM as shown in Table 3 were conducted individually using fresh uncoated solid tungsten carbide cutting tools (Make: Addison & Company Limited, Chennai, India) for each experiment. The tool has an overall length of 50 mm, diameter of 4 mm with two flutes and a helix angle of 30°.

**Figure 2:**
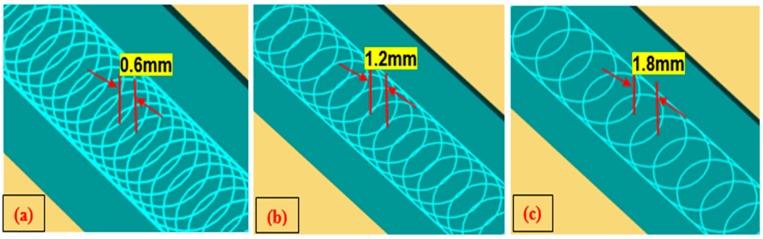
Trochoidal tool path step simulation at (**a**) s_tr_ = 0.6 mm (**b**) s_tr_ = 1.2 mm (**c**) s_tr_ = 1.8 mm.

**Figure 3:**
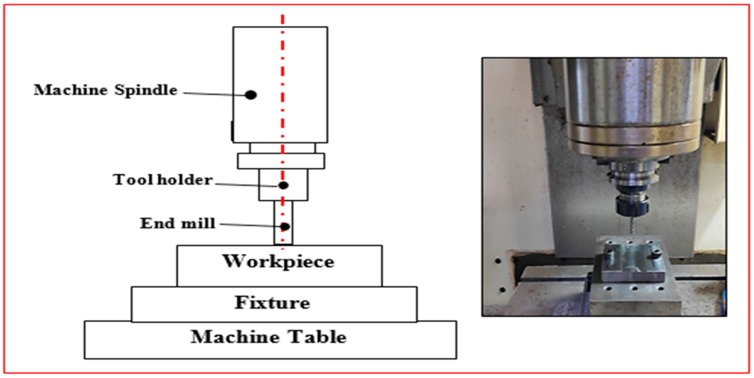
Experimental setup details.

In trochoidal milling, cutting tool is subjected to gradual milling by consecutive continuous circles and simultaneous forward moments. The trochoidal machining module in (NX.10, Siemens, Plano, TX, USA) CAM software was used to perform the trochoidal trajectory simulation as shown in Figure 2. In this study trochoidal step (S_tr_) parameter is a function of diameter of the tool. Therefore, for tool safety purposes, step values were taken less than 50% of tool diameter which is in the range 0.6 to 1.8 mm and loop diameter is automatically adjusted by CAM software based on the cavity. Trochoidal trajectory simulation was applied to machine the narrow slot cavity (75 mm × 30 mm) cross section. Trochoidal steps were taken as 0.6 mm, 1.2 mm and 1.8 mm. The milling operation was carried out in the direction of width of the workpiece. The experimental setup details are shown in Figure 3.

### Measurement of Output Responses

**Figure 4:**
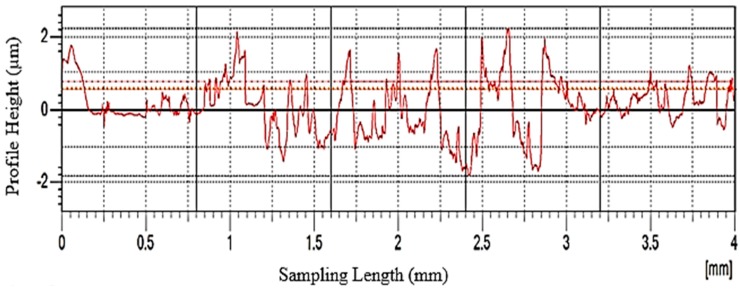
Surface roughness measurement graph.

**Figure 5:**
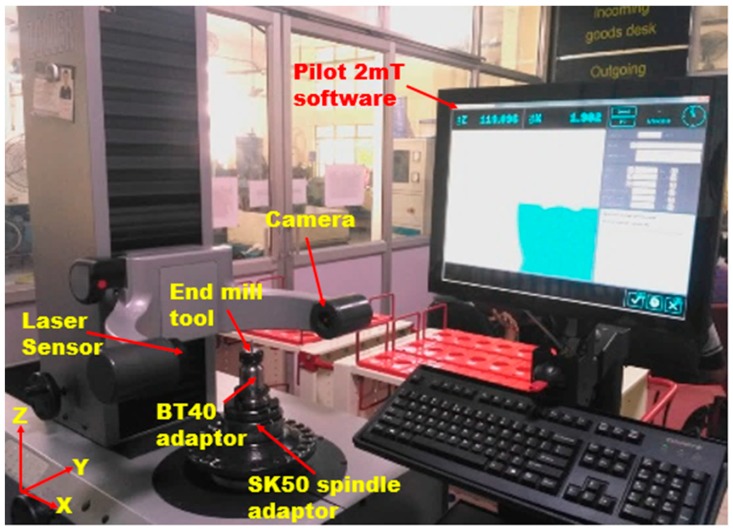
Zoller Junior Plus tool pre-setter used for dish angle measurement.

The surface roughness for each specimen was measured in the bottom of machined surface using a surface roughness testing machine (Model: Surfcom 1400 G, ACCRETECH, SIEMITSU, Tokyo, Japan) with 0.8 mm of sampling length and 4 mm of cut-off length. Specification of the roughness measuring instrument used are as follows: type: contact stylus instrument, stylus arm length of 60 mm and stylus radius of 2 μmR (60° conical diamond tip size), stylus measuring force of 0.75 mN and scanning rate of 1.5 mm/s. The detector has maximum stroke of 800 μm and the resolution is 0.1 nm in vertical range. Tracing driver column up/down speed is 10 mm/s. The accuracy of machine is 3 nm resolution at 0.2 mm and 15 nm resolution at 1mm in vertical range. An example of the surface roughness measurement graph obtained from ‘‘Accretech’’ software during the measurement is shown in Figure 4. The measurements were consequently repeated thrice at different locations along the feed direction of end milled surface and the arithmetic average value of surface roughness (R_a_), root mean square roughness (R_q_) and ten-point average height (R_z_) was noted as shown in Table 4. The initial cutting tool geometrical characteristics were determined using 2D and 3D measurements. The tool wear was examined by Vision measuring system (VMS) after performing milling operations. Surface roughness image and tool wear image were captured using Optive Lite Model (OLM) 3020 Vision with a color of CCD camera of 1/3 inches high resolution capacity with least count of 1μm integrated with LED stage light and ring light with field view of 30X to 180X magnification. The images were processed using VMS 3.1 software (Hexagon Manufacturing Intelligence, Noida, India). The Zoller Junior Plus (Zoller Inc., Deutschland, Germany) tool pre-setter as depicted in Figure 5 was utilized to calibrate the dish angle deviation. The measuring range of the pre setter is 420 mm in Z-axis and 210 mm in X-axis. The positioning accuracy of horizontal and vertical axis are ±0.003 mm and ±0.005 mm, respectively. The ZOLLER SK50 (steep taper 50, Zoller Inc., Deutschland, Germany) high-precision spindle with its concentricity of 0.002 mm used for indexing for picking up attachment holders for tool calibration. The maximum safe load on the table is 50 kg and maximum tool length and diameter is 320 mm and 620 mm, respectively. The chip set camera type (charge couple device monochrome model) and lighting system is 7 × 6 mm with 12 LEDs of red color for cutting edge calibration. 

**Figure 6:**
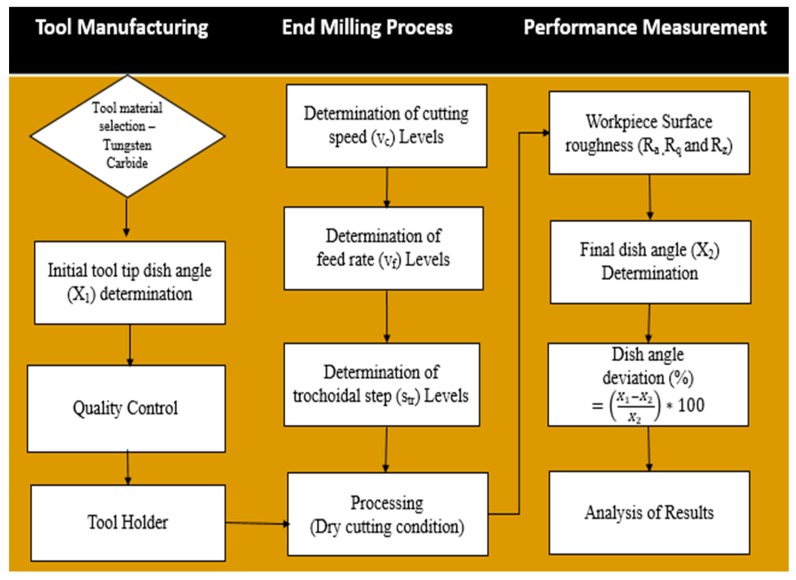
The experimental design procedure for cutting tool performance determination.

The dish angle value for the given set of runs were calibrated by Zoller tool pre-setter ‘‘Pilot 2mT’’ software system before and after machining. The initial dish angle (X_1_) is 1.55° for all the tools. The dish angle deviation was calculated using Equation (1):

(1) $$\mathrm{Dish}\;\mathrm{angle}\;\mathrm{deviation}\;\left(\%\right)=\left(\frac{X_{1}-X_{2}}{X_{2}}\right)\times 100$$

where, X_1_ is the dish angle before machining of the tool and X_2_ is the dish angle after machining of the tool. The experimental design procedure for the performance of tool determination is shown in Figure 6.

## 3. Response Surface Methodology (RSM) Experimental Design Matrix

RSM is a highly established technique for formulating the estimation models that rely on experimental observations or physical experiments. RSM is extensively used for optimization and development of mathematical models, which define the interdisciplinary relations of the process parameters and responses. The procedure for RSM follows six steps. The first step is to define the dependent responses and independent parameter. Next, the design of experiments orientation plan was formed with the independent factors based on CCD. Then, the appropriate multiple regression analysis was carried out [22]. The identification of significance factors and interactions using statistical analysis (ANOVA) follows. Finally, a confirmation test was performed to justify the developed model, after which the decision for the model’s acceptance or rejection was taken. Here, the dependent parameters taken were cutting speed, feed rate and trochoidal step, which are numeric, while the independent responses considered were surface roughness and dish angle. The measured output values are shown in Table 4.

Equations (2) and (3) represent the 1st and 2nd order developed mathematical correlation among the data sets, respectively [23,24], which was used for developing the empirical models relationships of the data that implies the best possible accuracy towards prediction:

(2) $$X_{i}=d_{0}+\;d_{1}x_{1}+\;d_{2}x_{2}+\;\ldots \ldots \;+\;d_{n}x_{n}$$

(3) $$X_{i}=d_{0}+\;\sum_{i=1}^{k}d_{i}x_{i}+\;\sum_{i=1}^{k}d_{i}x_{i}^{2}+\;\sum_{i=1}^{k}\sum_{j=1_{i<j}\;}^{k}d_{\mathrm{ij}}x_{i}x_{j}\;\pm \epsilon$$

where $X_{i}$ represents output responses, i.e., surface roughness(R_a_) and Dish angle; $d_{0}$ is a constant term, $d_{1},d_{2}\ldots \ldots .d_{n}$ in Equation (3) represents the coefficients of linear terms, while $d_{i},d_{\mathrm{ii}},\;d_{\mathrm{ij}}$ in Equation (4) denote the coefficients of linear terms, square terms and interaction terms, respectively; $x_{i}$ represents the input parameter i.e, Cutting speed (v_c_), federate (v_f_) and Trochoidal step (s_tr_). 

### 3.1. Developing Mathematical Relationships and *Regression Analysis*

The 2nd order polynomial quadratic model also known as regression model, describes the system behavior. Nonlinearity in Equation (3) is changed into its linear form using logarithmic transformation in order to generate the regression models. Design expert software version 11.0 (Stat-Ease, Inc., Minneapolis, MN, USA) was employed to determine the coefficients of response surface regression model in an empirical form. All the main parameters and its interaction parameters may not lead to vital consequences on the machining performance. In order to determine the significance of parameter ANOVA was utilized.

Based on the ANOVA results, the most significant process parameters were observed, and these parameters were incorporated on the final mathematical model relationships. Thus, obtained mathematical model relationships are listed below in Equations (4) and (5):

Ra = 0.3980 + 0.0172 × v_c_ + 0.0936 × v_f_ + 0.0447 × s_tr_ + 0.0050 × v_c_ × v_f_ + 0.0050 × v_c_ × s_tr_ + 0.0075 × v_f_ × s_tr_ + 0.0240 × v_c_^2^ + 0.0140 × v_f_^2^ + 0.0140 × s_tr_^2^ (4)

Dish Angle = 1.45 + 0.0530 × v_c_ − 0.0630 × v_f_ + 0.0310 × s_tr_ + 0.0113 × v_c_ × v_f_ + 0.0038 × v_c_ × s_tr_ + 0.0213 × f_z_ × s_tr_ − 0.0005 × v_c_^2^ − 0.0205 × v_f_^2^ − 0.0105 × s_tr_^2^ (5)

### 3.2. Evaluating the Correctness of the Empirical Relationship

The potential of the obtained empirical model is evaluated by ANOVA. Table 4 and Table 5 represent the ANOVA results for surface roughness and dish angle, respectively. The value of F indicates the significance of model. In Table 5 and Table 6, the value of p is greater than F and less than 0.0001, which implies that the developed models are vital [25]. Similarly, the effect of individual input terms (v_f_ × s_tr_) found to be significant for Ra and (v_c_ × v_f_ × s_tr_) found to be significant for dish angle and its interaction terms and 2nd order terms were found to be not significant for all the two output responses. Lack of fit value is smaller, and hence, it is not significant as desired.

**Figure 7:**
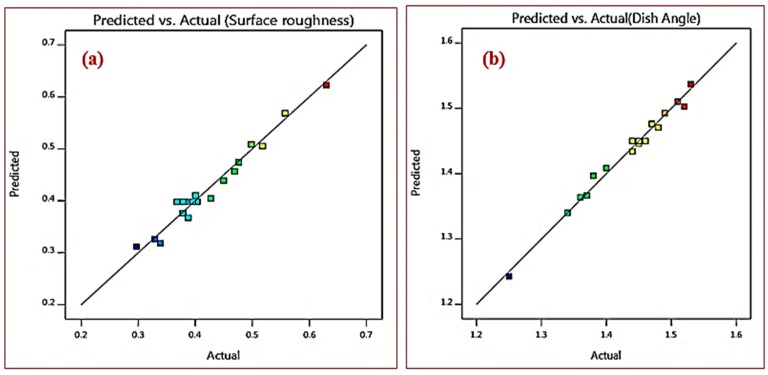
Prediction versus Actual correlation graphs (**a**) surface roughness (**b**) dish angle.

The obtained models possess high value of coefficient determination (R^2^) and adequate precision (AP). The obtained values are: R^2^ = 0.9687 and AP = 22.203 for surface roughness; and R^2^ = 0.9844 and AP = 35.64 for dish angle, which implies the goodness of fit of the models for the prediction of experimental results. The value of R^2^ adjusted is 0.9405 and 0.9705 for surface roughness and dish angle, respectively, which are higher, and denotes that higher importance of the developed model. R^2^ (predicted) and R^2^ (adjusted) are also in best agreement with each other. The low value of coefficient of variation (Cv) is 4.67 and 0.8132 for surface roughness and dish angle, respectively, which indicates the conducted experiments are reliable with high precision. Figure 7 shows the relationship between the experimental and predicted value. Each observed values of output responses of the AISI D3 tool steel samples were compared with an actual and predicted value obtained from empirical model, and its corresponding correlation plots. The ‘R^2^’ values for the obtained empirical relationship models seem to be within the range which implies that higher correlation persists between the predicted values and the actual values.

## 4. Results and Discussion

### 4.1. Effect of Process Parameter on Surface Roughness

Surface irregularities cannot be eliminated in any kind of machined surface. These irregularities can be examined by means of 2D roughness parameters (R_a_, R_q_, R_z_, R_sm_, R_t_, R_sk_ and R_ku_ etc.). In this current investigation, surface roughness was measured based on the value of Ra. Ra remains useful as a general guideline of surface texture ensuring uniformity in measurement of multiple surface. In addition, R_q_ and R_z_ was taken into consideration for assessing the influence of other amplitude parameters on surface texture of the machined surface. R_q_ denotes the root mean square average of the profile heights over the evaluation length and R_z_ is the average value of the absolute values of the heights of five highest profile peaks and the depths of five deepest valleys within the evaluation length. The rest of amplitude parameters, R_sk_, R_ku_, R_sm_ and R_t_, were not measured in the current study.

**Figure 8:**
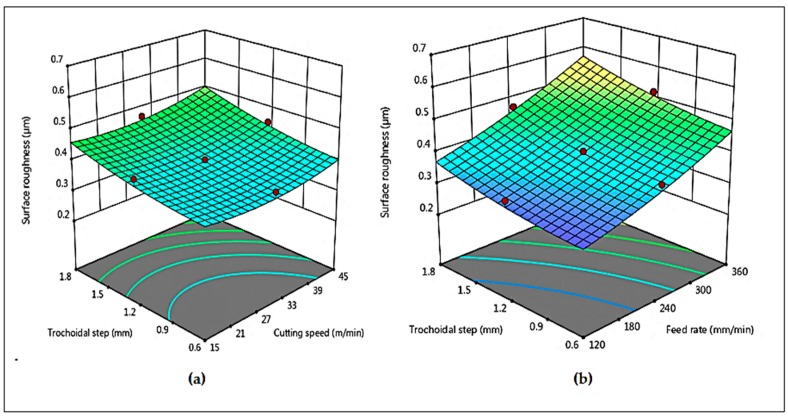
Combined effects of (**a**) v_c_ and s_tr_ on R_a_ (**b**) v_f_ and s_tr_ on R_a_.

The 3D surface plots act as an effective tool for investigating the behavior of responses with respect to two factors. In these plots, the dependent response is generally assigned to Z-axis where the independent factors are assigned to X and Y-axis. Figure 8a shows the surface plots of the mean Ra with respect to v_c_, v_f_ and s_tr_. From Figure 8a it is evident from the plot that mean Ra increases when increasing trochoidal step but decreases with increase in cutting speed. This can be interpreted by the fact that as the cutting speed increases, friction between cutting tool and work piece has been reduced that leads to suppression of built up edge (BUE) formation. On the other side increasing trochoidal step load on the tooltip is high which leads to increasing cutting force, thus causing poor surface roughness [10]. Figure 8b shows that mean Ra is increased with increase in feed rate and trochoidal step. This phenomenon may be explained by the fact that physical impression on the machined surface is forming of laces. 

**Figure 9:**
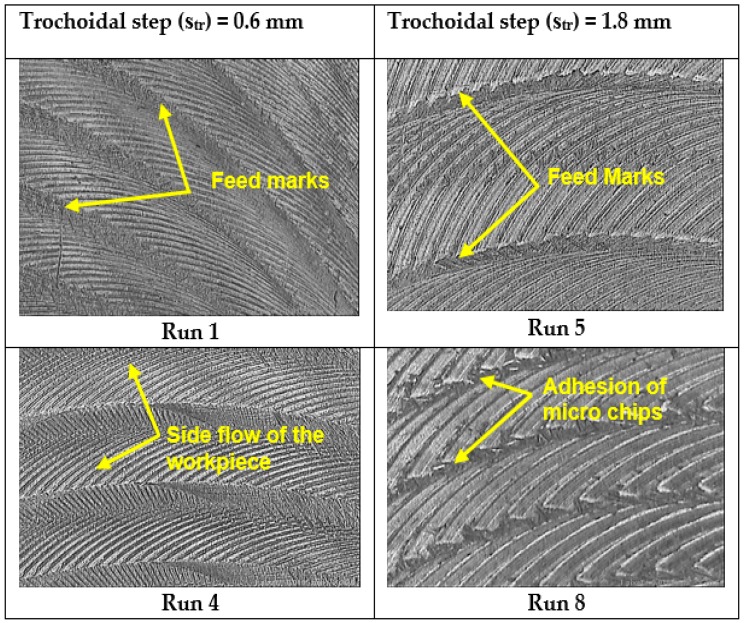
Effect of trochoidal step on surface roughness using Vision measuring system (corresponding to run numbers in Table 4).

The intensity of the appearance over the machined surface keep on increasing as the feed rate and trochoidal step is increased as shown in Figure 9. Additionally, the geometry of the tool influences the surface roughness. During milling operation with time, the cutting tool undergoes wear, which tends to change the tool profile during the operation. Similar results on surface roughness with respect to feed rate in end milling process of AISID2 steel was reported [26]. In addition, the effect of R_q_ and R_z_ were taken in to account. From Table 4 (Run 1 and Run 5), it can be observed that the trochoidal step has a predominant influence on the R_q_ and R_z_ parameter. The R_q_ value increases distinctly from 0.3135 μm to 0.4130 μm and R_z_ value increased from 2.0018 μm to 2.8704 μm when trochoidal step varying from 0.6 mm to 1.8 mm with respect to constant cutting speed of 15 m/min and feed rate of 120 mm/min. Similarly, for run 5 and run 8, the same behavior was observed for both R_q_ and R_z_ parameter. 

### 4.2. Effect of Process Parameter on Dish Angle

**Figure 10:**
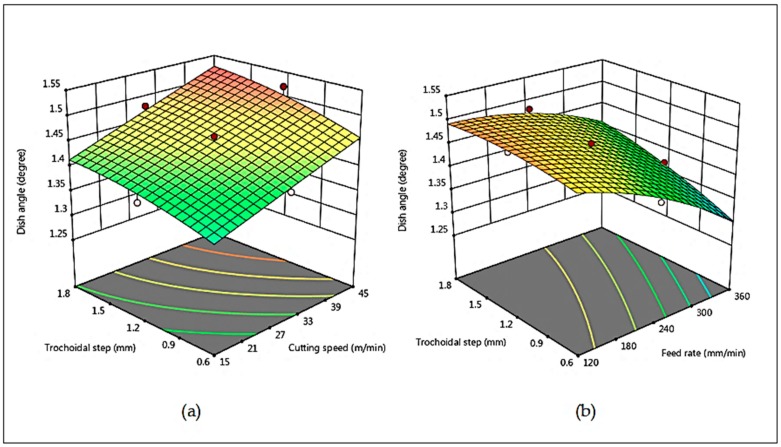
Combined effects of (**a**) v_c_ and s_tr_ on Dish angle (**b**) v_f_ and s_tr_ on Dish angle.

**Figure 11:**
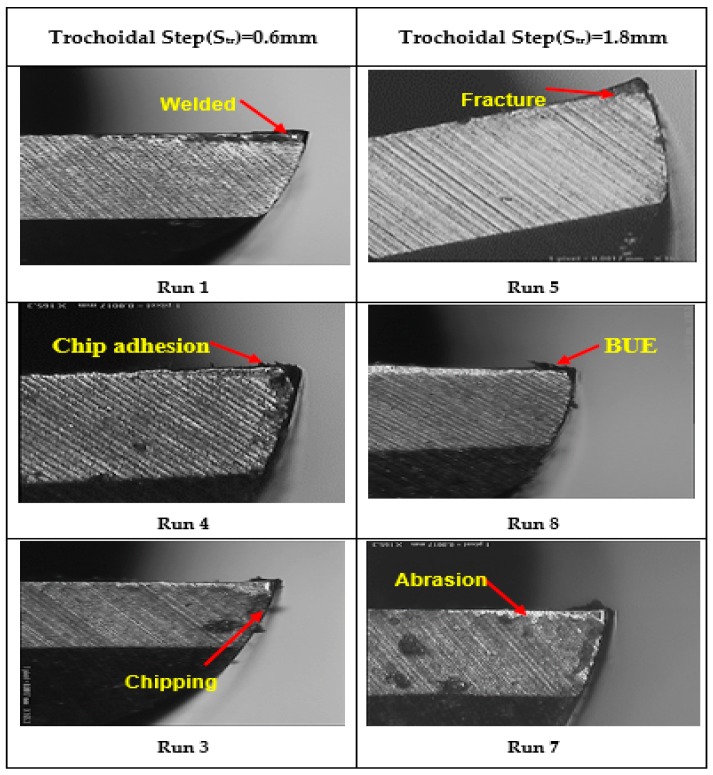
Effect of trochoidal step on dish angle (Tool wear) using Vision measuring system (corresponding to run numbers in Table 4).

Figure 10 reveals the 3D surface interaction plots for the dish angle in terms of v_c_, v_f_ and s_tr_, respectively. Figure 10a describes that the dish angle increases when cutting speed and trochoidal step increases. In trochoidal milling, the cutting tool is not always engaged with the workpiece for a period of time, as the material is subjected to gradual milling by consecutive continuous circles. Hence, the load or stress on the cutting tool is highly reduced due to the increase in trochoidal step with the increase of cutting speeds. These can be interpreted by the fact that due to increase in trochoidal step, the tool exhibits good heat dissipation. Therefore, it leads to a lower tool wear and an increased tool life [16]. From Figure 10b it is observed that the dish angle decreases with increase in feed rate and trochoidal step. This phenomenon may be explained by the fact that increasing trochoidal step built up edge is formed on the tool as shown in Figure 11. This may be the reason for the decrease in sharpness of cutting tool that tends to increase the tool wear resulting in the decrease of dish angle. Meanwhile, increase in feed rate is influenced with effect of high strain-hardening due to the plastic deformation and simultaneous increase in tool wear [26]. 

### 4.3. Multi Objective Optimization

**Figure 12:**
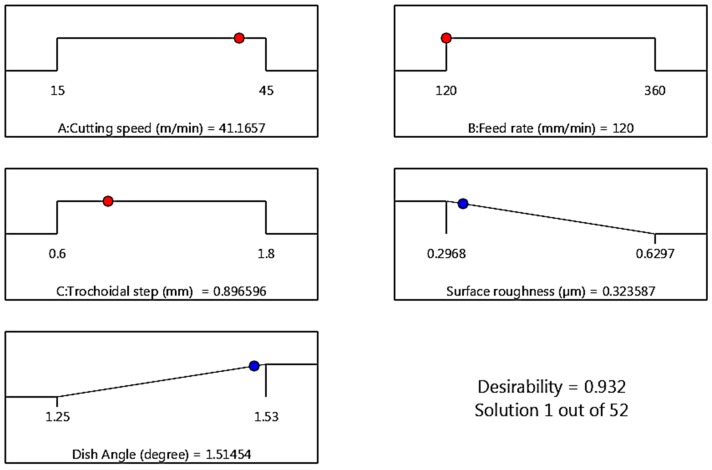
Ramps of numerical optimization.

**Figure 13:**
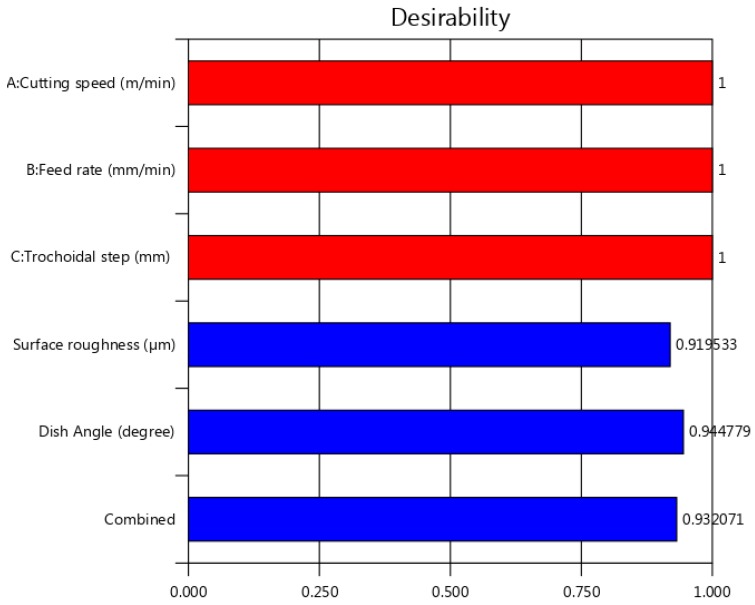
Two-dimensional (2D) composite desirability histogram plot.

The desirability approach is an optimization tool, which is a widely utilized technique in the industries for the multi-objective problem to determine the optimum values for the input process parameters and was proposed by Derringer and Suich [27]. The value of desirability varies between 0 and 1, which depends on the closeness of the output performance. To evaluate the desirability value of this experiment, Design Expert software version 11.0 package was used. Surface roughness and dish angle were optimized by the use of a set of values obtained from RSM. The ramp of numerical optimization graph and 2D composite desirability histogram plot of desirability are shown in Figure 12 and Figure 13 respectively. The ramp function shows the desirability values for each variable and each output response, as well as the composite desirability. Input parameter set or output quality prediction on a specific quality characteristic is quantified as per the dots on ramps. Each dot’s height signifies the desirability of the output quality response [27]. The nearest optimum region has an overall composite desirability value of 0.932, designating the closeness of the target value. The multiple regression model has been developed for the output responses (Ra, Dish angle) and was verified with the obtained experimental values, and these values were correlated with the results of confirmation experiments. The confirmation experiments were carried out thrice with optimum input process parameters of 41 m/min of v_c_, 210 mm/min of v_f_ and 0.9 mm of s_tr_. The confirmatory test and the predicted and obtained values of the output responses based on the optimization approach are shown in Table 7. 

It is understood from Table 7 that, the average of two output responses, mean surface roughness (0.344 μm) and dish angle (1.49°) of the experimental results seems to be very closer with RSM predicted values and its deviation is much smaller, which authenticate the predicted models is vital within the range of input parameters levels.

## 5. Conclusions

In this research work, experimental investigation and optimization studies in end milling operation on AISID3 cold work tool steel were performed using a trochoidal tool path to determine the effects of the cutting speed, feed rate and trochoidal step on the output responses. 20 trials for three input variables and three levels were successfully performed using face centered CCD of RSM. The 2nd order polynomial models were generated to predict the Ra and dish angle using RSM. The following inferences have been derived from the proposed work:

1. The developed mathematical modeling relationships of surface roughness and dish angle agreed well with the experimental results, since the error between the experimental and predicted value is within 6.10%, and 1.33%, respectively. The high rating of determination coefficients (R^2^) value prove their credibility.
2. The ANOVA studies substantiate that the most influence process parameter affecting the surface roughness is feed rate and trochoidal step, and the parameter dish angle is influenced by all the three-input parameters based on F-ratio value.
3. The formation of laces and adhesion of microchips on the machined surface leads to decrease the surface finish.
4. From the tool wear studies determined by the vision measurement system, it was concluded that the built-up-edge, chipping, abrasion and fracture leads to reduction in the dish angle. It was also concluded that the higher dish angle deviation was observed at a lower cutting speed, lower trochoidal step and higher feed rate.
5. Desirability based multi-objective optimization approach revealed that an optimum process parameter setting of 41m/min of v_c_, 210 mm/min of v_f_ and 0.9mm of s_tr_. It is summarized that the decrease in feed rate with increase in cutting speed and trochoidal step improve the output quality characteristics.

The results and conclusions obtained from this research will be more beneficial and advantageous for the researchers and machine tool industries for choosing optimum parameter setting for attaining desired surface finish and dish angle using trochoidal tool path, which is considered a novel parameter in end milling process within the range of limit considered for this study. 

Moreover, the obtained optimum level of process parameter will improve the quality of machined parts, thereby leading to substantial savings in the tool cost and improve the productivity.

## Figures and Tables

**Table 1:** Chemical composition in wt% of AISID3 cold work tool steel.

Element	Carbon (C)	Silicon (Si)	Chromium (Cr)	Manganese (Mn)	Nickel (Ni)	Vanadium (V)	Iron (Fe)
Content (wt%)	2.1	0.3	11.5	0.4	0.31	0.25	Balance

**Table 2:** AISI D3 cold work tool steel mechanical properties.

Workpiece Materials	Mechanical Properties of D3 Cold Work Tool Steel				
	Hardness, (HRC)	Density, (kg/cm^3^)	Tensile Strength, (N/mm^2^)	Yield Strength, (N/mm^2^)	Heat Conductivity, (W/mK)
AISI D3	30	7.7	970	850	20

**Table 3:** Process factors and their coded values.

Factors	Symbol	Units	Coded Values		
			(−1)	(0)	(+1)
Cutting speed	v_c_	m/min	15	30	45
Feed rate	v_f_	mm/min	120	240	360
Trochoidal step	s_tr_	mm	0.6	1.2	1.8

**Table 4:** Experimental results for the end milling operation.

Run	Input Parameters			Output Responses				
	v_c_ (m/min)	v_f_ (mm/min)	s_tr_ (mm)	*Ra* (μm)	R_q_ (μm)	R_z_ (μm)	Dish Angle (degree)	Dish Angle Deviation (%)
1	15	120	0.6	0.2968	0.3135	2.0018	1.42	8.38
2	45	120	0.6	0.3292	0.3592	2.4050	1.51	2.5
3	15	360	0.6	0.4759	0.5070	3.3420	1.25	19.35
4	45	360	0.6	0.4983	0.5288	3.6948	1.36	12.25
5	15	120	1.8	0.3783	0.4130	2.8704	1.45	6.45
6	45	120	1.8	0.4006	0.4309	3.5797	1.53	1.29
7	15	360	1.8	0.5574	0.6017	4.9839	1.34	13.54
8	45	360	1.8	0.6297	0.7478	5.6560	1.47	5.16
9	15	240	1.2	0.4271	0.5324	3.7702	1.38	10.96
10	45	240	1.2	0.4494	0.4812	2.8073	1.52	1.93
11	30	120	1.2	0.3387	0.3963	2.1260	1.49	3.87
12	30	360	1.2	0.5178	0.6138	4.2041	1.37	11.61
13	30	240	0.6	0.3875	0.4334	2.6660	1.40	9.67
14	30	240	1.8	0.4690	0.5012	3.1440	1.48	4.51
15	30	240	1.2	0.3949	0.4237	2.4030	1.45	6.45
16	30	240	1.2	0.3824	0.4131	2.2032	1.44	7.09
17	30	240	1.2	0.4032	0.4631	2.4601	1.46	5.8
18	30	240	1.2	0.3786	0.4284	2.3031	1.45	6.45
19	30	240	1.2	0.3678	0.4057	2.4216	1.46	5.8
20	30	240	1.2	0.3956	0.4317	2.6856	1.44	7.09

**Table 5:** Analysis of variance (ANOVA) table for surface roughness.

Source	SS	d.f	MS	F-Value	p-Value Prob > F	Remarks
Model	0.1213	9	0.0255	34.38	<0.0001	significant
v_c_	0.0029	1	0.2165	7.52	0.0208	
v_f_	0.0875	1	0.0013	223.18	<0.0001	
s_tr_	0.0200	1	0.0001	51.02	<0.0001	
v_c_ × v_f_	0.0002	1	0.0050	0.5100	0.4915	
v_c_ × s_tr_	0.0002	1	0.0002	0.5049	0.4936	
v_f_ × s_tr_	0.0005	1	0.0039	1.15	0.3092	
v_c_^2^	0.0016	1	0.0008	4.03	0.0726	
v_f_^2^	0.0005	1	0.0018	1.37	0.2694	
s_tr_^2^	0.0005	1	0.0000	1.37	0.2694	
Residual	0.0039	10	0.0001			
Lack of fit	0.0031	5		3.56	0.0947	not significant
Pure Error	0.0009	5				
Total	0.1252	19				
Standard Deviation	0.0198			R^2^	0.9687	
Mean	0.4239			Adjusted R^2^	0.9405	
Cv %	4.67			Predicted R^2^	0.8152	
				Adeq Precision	22.203	

**Table 6:** ANOVA table for Dish angle.

Source	SS	d.f	MS	F-Value	p-Value Prob > F	Remarks
Model	0.0861	9	0.0096	70.34	<0.0001	significant
v_c_	0.0281	1	0.0281	206.44	<0.0001	
v_f_	0.0397	1	0.0397	291.69	<0.0001	
s_tr_	0.0096	1	0.0096	70.63	<0.0001	
v_c_ × v_f_	0.0010	1	0.0010	7.44	0.0213	
v_c_ × s_tr_	0.0001	1	0.0001	0.8268	0.3846	
v_f_ × s_tr_	0.0036	1	0.0036	26.55	0.0004	
v_c_^2^	5.682 × 10^−7^	1	5.682 × 10^−7^	0.0042	0.9498	
V_f_^2^	0.0012	1	0.0012	8.46	0.0156	
s_tr_^2^	0.0003	1	0.0003	2.21	0.1680	
Residual	0.0014	10	0.0001			
Lack of fit	0.0010	5		2.40	0.1792	not significant
Pure Error	0.0004	5				
Total	0.0875	19				
Standard Deviation	0.0117			R^2^	0.9844	
Mean	1.43			Adjusted R^2^	0.9705	
Cv. %	0.8132			Predicted R^2^	0.9067	
				Adeq Precision	35.6438	

**Table 7:** Optimum parameter level and output response.

Optimum Run	Input Parameters			Ra (μm)		Dish Angle (°)	
	v_c_	v_f_	s_tr_	Value	%[Error]	Values	%[Error]
Optimum (RSM)	41	120	0.9	0.323	6.10	1.51	1.34
Optimum (Actual)	41	120	0.9	0.344	-	1.49	-

## Abbreviations

**Notation**	
AISI	American Iron & Steel Institute
ANOVA	Analysis of variance
HRC	Hardness measured with the Rockwell test for hard materials
CAM	Computer aided manufacture
RSM	Response surface methodology
BUE	Built up edge
CCD	Central composite design
MRR	Material removal rate
SS	Sum of Squares
MS	Mean Square Symbols
VMS	Vision measuring system
d.f	Degree of freedom
**Symbol**	
s_tr_	The trochoidal step means the distance between adjacent centers of revolution path (mm)
v_c_	Cutting speed (m/min)
v_f_	Feed rate(mm/min)
R_a_	Surface roughness (μm)
R_z_	Ten point average height (μm)
R_q_	Root-mean-square roughness (μm)
R_sk_	skewness
R_ku_	kurtosis
R_sm_	Mean spacing between profile peaks at the mean line
X_i_	Estimated output response
x_i_	Represents the input parameter
d_0_	Free term of regression equation
d_i_	Coefficients of linear terms
d_ij_	Coefficients of square terms
∈	Experimental error
d_1_, d_2_…d_n_	coefficients of linear terms
X_1_	Before machining of the tool
X_2_	After machining of the tool

## References

[1] Kuczmaszewski J., Zaleski K., Matuszak J., Palka T., Madry J. (2017). Studies on the effect of mill microstructure upon tool life during slot milling of Ti6Al4V alloy parts. Maintenance Reliab..

[2] Polvorosa R., Suarez A., Lopez de Lacalle L.N., Cerrillo I., Wretland A., Veiga F. (2017). Tool wear on nickel alloys with different coolant pressures: Comparison of Alloy 718 and Waspaloy. J. Manuf. Process..

[3] Chen W.J., Hsu C.C., Yang Y.L. (2014). Improving roughness quality of end milling Al 7075-T6 alloy with Taguchi based multi objective quantum behaved particle swarm optimisation algorithm. Mater. Res. Innov..

[4] Wang M.-Y., Chang H.-Y. (2004). Experimental study of surface roughness in slot end milling AL2014-T6. Int. J. Mach. Tools Manuf..

[5] Mahesh G., Muthu S., Devadasan S.R. (2015). Prediction of surface roughness of end milling operation using genetic algorithm. Int. J. Adv. Manuf. Technol..

[6] Ren J.X., Zhou J.H., Wei J.W. (2015). Optimization of Cutter Geometric Parameters in End Milling of Titanium Alloy Using the Grey-Taguchi Method. Adv. Mech. Eng..

[7] Wang Y.-C., Chen C.-H., Lee B.-Y. (2014). Analysis model of parameters affecting cutting performance in high-speed machining. Int. J. Adv. Manuf. Technol..

[8] Topal E. (2009). The role of stepover ratio in prediction of surface roughness in flat end milling. Int. J. Mech. Sci..

[9] Gologlu C., Sakarya N. (2008). The effects of cutter path strategies on surface roughness of pocket milling of 1.2738 steel based on Taguchi method. J. Mater. Process. Technol..

[10] Li H., Peng F.Y., Tang X.W., Xu J.W., Zeng H.H. (2017). Stability prediction and step optimization of Trochoidal Milling. J. Manuf. Sci. Eng..

[11] Akhavan Niaki F., Pleta A., Mears L. (2018). Trochoidal milling: investigation of a new approach on uncut chip thickness modeling and cutting force simulation in an alternative path planning strategy. Int. J. Adv. Manuf. Technol..

[12] Ibaraki S., Yamaji I., Matsubara A. (2010). On the Removal of Critical Cutting Regions by Trochoidal Grooving. Precis. Eng..

[13] Deng Q., Mo R., Chen Z.C., Chang Z.Y. (2017). A new approach to generating trochoidal tool paths for effective corner machining. Int. J. Adv. Manuf. Technol..

[14] Uriarte L., Azcarate S., Herrero A., Lopez de Lacalle L.N., Lamikiz A. (2008). Mechanistic modelling of the micro end milling operation. Proc. Inst. Mech. Eng. B J. Eng. Manuf..

[15] Patil P., Polishetty A., Golberg M., Littlefair G., Nomani J. (2014). Slot Machining of TI6AL4V with Trochoidal Milling Technique. J. Mach. Eng..

[16] Wu S.X., Ma W., Li B., Wang C.Y. (2016). Trochoidal machining for high speed milling of pockets. J. Mater. Process. Technol..

[17] Palanisamy P., Rajendran I., Shanmugasundaram S. (2008). Prediction of tool wear using regression and ANN models in end-milling operation. Int. J. Adv. Manuf. Technol..

[18] Sivaraosa K.R., Milkey A.R., Samsudin A.K., Dubey Kidd P. (2014). Comparison between Taguchi method and response surface methodology (RSM) in modelling CO2 laser machining. Jordan J. Mech. Ind. Eng..

[19] Mia M. (2017). Multi-response optimization of end milling parameters under through-tool cryogenic cooling condition. Measurement.

[20] Abou-El-Hossein K.A., Kadirgamaa K., Hamdi M., Benyounis K.Y. (2007). Prediction of cutting force in end-milling operation of modified AISI P20 tool steel. J. Mater. Process. Technol..

[21] Calıskan H., Kurbanoglu C., Panjan P., Kramar D. (2013). Investigation of the performance of carbide cutting tools with hard coatings in hard milling based on the response surface methodology. Int. J. Adv. Manuf. Technol..

[22] Rajeswari B., Amirthagadeswaran K.S. (2017). Experimental investigation of machinability characteristics and multi response optimization of end milling in aluminium composites using RSM based grey relational analysis. Measurement.

[23] Mohammed Iqbal U., Senthil Kumar V.S., Gopala Kannan S. (2016). Application of Response Surface Methodology in optimizing the process parameters of Twist Extrusion process for AA6061-T6 aluminum alloy. Measurement.

[24] Chauhan S.R., Dass K. (2012). Optimization of Machining Parameters in Turning of Titanium (Grade-5) Alloy Using Response Surface Methodology. Mater. Manuf. Process..

[25] Ravi Kumar K., Sreebalaji V.S. (2015). Desirability based multi objective optimisation of abrasive wear and frictional behaviour of aluminium (Al/3.25Cu/8.5Si)/fly ash composites. Tribol. Mater. Surf. Interfaces.

[26] Koshy P., Dewes R.C., Aspinwall D.K. (2002). High speed end milling of hardened AISI D2 tool steel (58 HRC). J. Mater. Process. Technol..

[27] Mohammed Iqbal U., Senthil Kumar V.S. (2014). Modeling of twist extrusion process Parameters of AA6082-T6 alloy by response surface approach. Proc. Inst. Mech. Eng. B J. Eng. Manuf..

